# Effects of Mobile-Based AI-Enhanced Health Counseling on Protection Motivation, Self-Care Behaviors, and Periodontal Treatment Outcomes in Patients With Periodontitis: Randomized Controlled Trial

**DOI:** 10.2196/78211

**Published:** 2025-10-28

**Authors:** You-Jie Hu, Pei-Chen Lin, Pei-Chao Lin, Chiung-Lin Huang, Chih-Chang Chen, Koichiro Matsuo, Chien-Hung Lee, Hsiao-Ling Huang

**Affiliations:** 1 Department of Oral Hygiene, College of Dental Medicine, Kaohsiung Medical University, 100 Shih-Chuan 1st Road, Kaohsiung, 807378, Taiwan, 886 7-3121101 ext 2159; 2 Department of Medical Research, Kaohsiung Medical University Hospital, Kaohsiung Medical University, Kaohsiung, Taiwan; 3 School of Nursing, College of Nursing, Kaohsiung Medical University, Kaohsiung, Taiwan; 4 Master Program of Long-Term Care in Aging, College of Nursing, Kaohsiung Medical University, Kaohsiung, Taiwan; 5 Center for Neurotechnology and Assistive Technology, Kaohsiung Medical University, Kaohsiung, Taiwan; 6 Center for Long-Term Care Research, Kaohsiung Medical University, Kaohsiung, Taiwan; 7 Division of Periodontics, Department of Dentistry, Kaohsiung Medical University Chung-Ho Memorial Hospital, Kaohsiung, Taiwan; 8 School of Dentistry, College of Dental Medicine, Kaohsiung Medical University, Kaohsiung, Taiwan; 9 Department of Oral Health Sciences for Community Welfare, Graduate School of Medical and Dental Sciences, Institute of Science Tokyo, Tokyo, Japan; 10 Department of Public Health, College of Health Science, Kaohsiung Medical University, Kaohsiung, Taiwan; 11 Research Center for Precision Environmental Medicine, Kaohsiung Medical University, Kaohsiung, Taiwan

**Keywords:** artificial intelligence, health behavior, motivation, periodontal diseases, quality of life

## Abstract

**Background:**

Patients’ protection motivation (PM) and self-care behaviors are essential for sustaining oral hygiene adherence and long-term periodontal health.

**Objective:**

This study aimed to evaluate the effects of artificial intelligence (AI)-assisted dental monitoring on PM, behavioral change, and periodontal treatment outcomes.

**Methods:**

Patients with periodontitis were randomly assigned to an AI group (n=32), an AI-assisted health counseling (AIHC) group (n=33), or a control group (n=33). All participants received nonsurgical periodontal treatment and oral hygiene instruction. The AI and AIHC groups additionally received AI-based monitoring and, for the AIHC group, personalized counseling for 3 months. PM (threat and coping appraisal), self-care behavior, plaque control record, probing pocket depth, and clinical attachment level were assessed at baseline and follow-ups. Generalized estimating equations were used to analyze the follow-up effects over time, with an intention-to-treat analysis for group comparisons.

**Results:**

Significant time effects were observed for threat appraisal and coping appraisal at 6 months (adjusted mean ratio 1.07 and 1.06, respectively), with no significant group-by-time interaction. At 6 months, both AI and AIHC groups showed greater improvements in self-care behavior (AI: adjusted regression coefficient [aβ] 0.32; AIHC: aβ 0.29) and plaque control record (AI: aβ −13.28; AIHC: aβ −23.22) compared to the control group. At 3 months, the AI and AIHC groups also demonstrated greater reductions in probing pocket depth (aβ −0.43 and −0.55, respectively) and clinical attachment level (aβ −0.44 and −0.51, respectively).

**Conclusions:**

AI-assisted monitoring improved self-care behaviors, plaque control, and periodontal clinical outcomes. Although PM constructs (threat appraisal and coping appraisal) improved similarly across all groups, the greater behavioral and clinical improvements observed in the AI-assisted groups highlight the potential of AI monitoring as an effective tool to enhance periodontal care. Incorporating AI technologies into routine practice may empower patients to take a more active role in managing their oral health and support more effective, patient-centered periodontal therapy.

## Introduction

Periodontitis affects between 20% and 50% of the global population [1] and is the sixth most common disease globally, with severe periodontitis affecting approximately 11.2% of individuals [2]. As of 2019, the global burden of severe periodontitis was estimated at approximately 1.1 billion cases [3]. The main cause of periodontitis is a chronic inflammatory reaction to microbial biofilms, leading to clinical manifestations such as discomfort, bleeding, increased tooth mobility, destruction of the attachment apparatus, and eventual tooth loss [4]. This damage affects chewing function, aesthetics, social interactions, and oral health–related quality of life. In addition, severe periodontitis may contribute to systemic diseases such as diabetes [5], cardiovascular diseases [6], dementia [7], and depression [8]. Poor oral hygiene causes a microbial biofilm to form, and the matrix of this film protects microbial cells against chemical and physical damage and hinders the eradication of pathogenic dental plaque [9]. The American Dental Association recommends that preventive care include brushing at least twice a day, flossing at least once a day, and visiting the dentist at least once per year [10].

Artificial intelligence (AI) applications can streamline care, perform laborious routine tasks, and enable dentists to spend additional time on patient health, reducing staffing requirements in dental clinics and facilitating personalized, predictive, preventive, and participatory dentistry [11]. One study using AI-based diagnostic support software for dental caries detection demonstrated that AI can increase dentists’ diagnostic accuracy [12]. AI-assisted dental monitoring is also used for the evaluation of clear aligners, enabling patients to remotely consult with dentists from home regarding their oral and orthodontic conditions, thereby promoting a positive doctor-patient relationship and increasing treatment efficiency [13].

Applying AI in home-based health care offers a cost-effective and convenient means to support patients with periodontitis. Our previous studies have demonstrated the benefits of AI-assisted monitoring on periodontal clinical outcomes such as probing pocket depth (PPD), clinical attachment level (CAL), and plaque control record (PCR), which are well-established and clinically meaningful indicators of periodontal health [14,15]. While improvements in clinical periodontal parameters are essential, behavioral factors—particularly patients’ motivation and self-care behaviors—play a crucial role in sustaining oral health. However, a meta-analysis has shown that traditional health education yields only limited effects on patient motivation and behavior change [16]. We hypothesized that AI-assisted monitoring could strengthen patients’ intrinsic motivation and promote better self-care behavior, thereby leading to superior clinical outcomes compared to conventional education. Therefore, this randomized controlled trial primarily aimed to evaluate the effects of AI-assisted monitoring on clinical periodontal outcomes, with PPD and CAL defined as the primary endpoints specified a priori. In addition, the study examined the intervention’s impact on oral hygiene behavior and psychological constructs based on protection motivation (PM) theory (ie, threat and coping appraisal) as secondary outcomes.

## Methods

### Study Design and Participants

A single-blind randomized controlled trial was conducted in this study. Patients diagnosed with periodontitis were recruited in person from the Division of Periodontics at Kaohsiung Medical University between 2020 and 2024. Eligible participants were those aged 35‐64 years who received a new diagnosis of periodontitis on the basis of the 2017 classification (Stages III and IV), with diagnosis determined by CAL [17]. Participants with at least 16 functional teeth and a minimum of 4 supporting zones (classified as Category A according to the Eichner index) [18] were included. Exclusion criteria comprised a history of periodontal treatment within the past 6 months, regular use of antibiotics or bisphosphonates, pregnancy or lactation, and the presence of systemic conditions such as diabetes, cancer, renal or hepatic failure, or cardiovascular disease.

An effect size calculation was performed on the basis of an earlier study comparing AI-assisted groups with a control group in terms of CAL. The sample size calculation accounted for a type I error of 0.05, a power of 0.80, and an effect size (Cohen *d*) of 0.74 [15,19]. Repeated-measures ANOVA with a within- and between-subjects interaction was conducted using G*Power (version 3.1.5; G*Power [20]). The minimum sample size was determined to be 30 participants per group. To provide a buffer against a 20% attrition rate, the final minimum sample size was set at 36 participants per group.

### Randomization and Blinding

A random number table was used to generate the random allocation sequence, assigning patients to 1 of 3 groups: AI (n=32), AI-assisted human counseling (AIHC; n=33), or control (n=33). Participants were randomized in a concealed manner into the 3 groups using a computer-generated algorithm implemented through Excel (Microsoft Corp) with an allocation ratio of 1:1:1. This process involved simple randomization without restrictions such as blocking. Allocation concealment was ensured by assigning the randomization process to a third-party staff member who was not involved in patient enrollment or data collection. To conceal the allocation sequence until interventions were assigned, sequentially numbered containers were used. The random allocation sequence was generated by the study coordinator, while the researcher enrolled participants and assigned them to interventions. Both the dentists providing periodontal treatment and the dental hygienist assessing PCR were blinded to group assignments.

### Dental Examination

A periodontist performed periodontal examinations using standard periodontal probes, dental mirrors, and periodontal assessment forms to evaluate 2 periodontal parameters: PPD and CAL. Two dental examiners underwent calibration for periodontal measurements following the procedure described by Hill et al [21]. The intrarater reliability for PPD, assessed using the intraclass correlation coefficient, was 0.97. Interrater reliability between the 2 examiners was also evaluated, with the intraclass correlation coefficient for PPD recorded at 0.84.

### Questionnaire Development

A structured questionnaire was used to collect data on demographic information, protective motivation, and self-care behaviors. To evaluate face and content validity, the questionnaire was reviewed by a panel of experts, resulting in a content validity index ranging from 0.96 to 1.00. A pilot test was conducted with a convenience sample of 10 patients diagnosed with periodontitis to ensure clarity and comprehensibility of the questionnaire. Based on feedback from the pilot test, revisions were made to improve item clarity and relevance.

### Oral Health Counselor Recruitment and Training

Dental hygienists were recruited to act as oral health counselors to complement the AI platform. They underwent a 4-hour training program that included topics such as periodontal disease, oral hygiene practices, communication techniques for counseling, AI system usage skills, intraoral assessment, and image interpretation via the AI platform. Throughout the intervention, research staff maintained continuous communication with the counselors to provide support and address any challenges related to the use of the AI system.

### Periodontal Treatment

Nonsurgical periodontal treatment was provided to all 3 groups by 1 periodontist. Each participant’s treatment was completed within 1‐2 months. This treatment adhered to the S3-level Clinical Practice Guidelines for the management of Stage I–III periodontitis, as established by the European Federation of Periodontology [22,23], and included full-mouth scaling, root planing, and oral hygiene education. A 30-minute one-on-one session was conducted by a dental hygienist, covering information on periodontitis, proper brushing techniques, interdental cleaning, and the use of oral hygiene aids. Standardized lecture slides were presented on a tablet to facilitate the participants’ understanding of the material. The instruction session also involved the use of an AI-assisted camera to help the participants evaluate the effectiveness of their cleaning after a plaque-disclosing agent had been applied. During the session, the participants were given a soft-bristled toothbrush, fluoride toothpaste, dental floss, an interdental brush, and assistance with how to select and use dental cleaning tools.

### Intervention

The AI and AIHC groups received a 30-minute training session on how to use the AI-assisted tool and navigate the interface of the DentalMonitoring (DentalMonitoring) app. To accommodate participants with varying levels of health and digital literacy, the DentalMonitoring intervention incorporated several user-friendly features, including voice-guided prompts, simplified visual instructions, and instructional videos embedded within the app. These design elements aimed to facilitate independent use and enhance engagement across diverse literacy levels.

Participants were provided with an activation link via email, granting free access to the DentalMonitoring app. The scanning procedure required the use of a cheek retractor, a scanbox, and a smartphone. Using this setup, patients captured intraoral photographs at home. The AI system then analyzed these images using a large dental database and provided feedback on oral hygiene and gingival status. Based on the results, the system suggested individualized oral hygiene strategies and treatment recommendations, enabling patients to monitor their periodontal condition in real time and adopt appropriate self-care behaviors. This AI-assisted DentalMonitoring intervention followed the methodology outlined in a previous study [14], which reported satisfactory interrater agreement between periodontists and the AI system (Cohen κ=0.8).

During the study, participants in the AI and AIHC groups conducted self-administered intraoral scans at home once per week for 3 months, with each scan taking approximately 5 minutes. On average, each participant completed 13 intraoral scans throughout the intervention period. Following each scan, the results were uploaded to the AI system, which generated feedback within 48 hours. The AI system was programmed to automatically deliver feedback messages based on the patient’s oral hygiene status, such as: “Your oral hygiene status is excellent, please keep up the good work,” or “Your oral hygiene status needs improvement, please enhance your cleaning efforts.” In addition, if a participant did not upload a scan within the designated timeframe, the system automatically sent reminder notifications through the platform. These automated reminders were delivered to all participants for every scheduled scan as part of the standard protocol.

In the AIHC group, participants received both AI assistance using DentalMonitoring and oral health counseling from a hygienist. The counselor assessed their oral hygiene based on the scanning results and provided personalized weekly counseling through the AI platform, including guidance on oral cleaning techniques and recommendations for cleaning tools. Participants’ questions were addressed through the text messaging feature of DentalMonitoring. The control group did not receive any AI intervention.

### Outcomes

#### Periodontal Parameters

The PPD and CAL [24] were recorded at 6 sites (mesial [buccal and lingual/palatal], distal [buccal and lingual/palatal], mid [buccal, and midlingual/palatal]) around each tooth except the third molar. The severity of PPD is classified as follows: 1‐3 mm indicates healthy gums, 4‐5 mm indicates mild periodontitis, 6‐7 mm indicates moderate periodontitis, and >7 mm indicates severe periodontitis. The severity of CAL is classified as follows: 0‐2 mm indicates healthy, 3‐4 mm indicates moderate, and >4 mm indicates severe periodontitis.

#### Plaque Control Record

An oral hygienist recorded the PCR [25] for each participant using tweezers, cotton balls, a plaque disclosing agent, and a standardized PCR chart. The disclosing agent was applied to stain all tooth surfaces, with each tooth divided into 6 segments. Each segment was scored as 1 if plaque was detected and 0 if no plaque was present. The PCR score was calculated as the percentage of plaque-positive surfaces out of the total number of assessed surfaces. Only natural teeth were included in the evaluation.

#### Self-Care Behavior

The self-care behavior assessment consisted of 5 questions. First, participants were asked how many times they brushed their teeth each day (once, twice, thrice, or 4 times or more); responses of thrice or more were scored as 1, and all other responses received a score of 0. Second, participants were asked how long they spent brushing their teeth (1, 1‐2, 2‐3, or >3 minutes); responses of more than 3 minutes were scored as 1, and all other responses were scored as 0. Third, participants indicated their primary brushing method, choosing from horizontal scrubbing, vertical scrubbing, circular brushing, rotating brushing, or a combination of horizontal and vertical brushing with the bristles positioned at a 45° angle to the gum line, known as the Bass brushing technique; the combination method was scored as 1, and all others as 0. Fourth, participants were asked whether they used a soft-bristled toothbrush, with “yes” scored as 1 and “no” scored as 0. Finally, participants indicated whether they used interdental brushes, with “yes” scored as 1 and “no” scored as 0. The total score for the self-care behavior assessment ranged from 0 to 5.

#### Protection Motivation

The PM scale [26] consists of threat appraisal (2 items, score range 2‐20) and coping appraisal (2 items, score range 2‐20). The threat appraisal items are “I think periodontitis is a serious disease” and “If I do not adopt behaviors for preventing periodontitis, my risk of developing periodontal disease will be high.” The coping appraisal items were “If I adhere to behaviors for preventing periodontitis, my oral health will improve” and “I can follow periodontal care instructions.” Each item is scored on a scale from 1 (not at all likely) to 10 (extremely likely). The total score ranges from 7 to 70, with a higher score indicating stronger behavioral motivation. The Cronbach α for the PM scale is 0.76, calculated from pilot study data.

### Data Collection

Data on PM, self-care behavior, and PCR were collected at 4 time points: baseline and the 1-month, 3-month, and 6-month follow-ups. PPD and CAL were collected at baseline and the 3-month follow-up. Participants completed a structured questionnaire at the Department of Periodontics clinic, Kaohsiung Medical University Hospital. The questionnaire was administered by trained research staff and took 10‐20 minutes to complete.

### Statistical Analysis

All statistical analyses were performed using STATA (version 13.0; StataCorp). Baseline differences in age among the 3 groups were assessed using 1-way ANOVA, while differences in sex and educational level were examined using chi-square tests. The Shapiro-Wilk test was used to assess the normality of continuous outcome variables. Variables that were not normally distributed were logarithmically transformed for parametric analysis.

Boxplots were used to illustrate the distribution of PPD and CAL at baseline and 3 months across the 3 groups, showing median, IQR, and outliers for visual comparison of changes and variability over time. We evaluated the linear trend of outcomes across increasing time points by assigning ordered scores to each time point and treating the time variable as continuous. Generalized estimating equations (GEEs) with an autoregressive correlation structure were used to assess group effects (AI and AIHC groups vs control), time effects, and interactions between groups and time points regarding threat appraisal, coping appraisal, self-care behavior, PCR, PPD, and CAL over a 6-month follow-up. GEE is a robust statistical method designed for analyzing repeated measures data. For nonnormally distributed outcomes, specifically threat appraisal and coping appraisal, GEE models were specified with a gamma distribution and a log link function to appropriately address data transformation and nonnormality. Exponentiated regression coefficients were calculated to represent mean ratios for the AI and AIHC groups compared to the control group. Main-effect GEE models were used to interpret the findings when interaction effects were not significant. All models were adjusted for covariates, including age, sex, and educational level. A 2-sided *P* value of <.05 was considered statistically significant. We used an intention-to-treat approach, including all participants with available follow-up data.

To address missing data, particularly due to attrition in the control group, we performed multiple imputation (MI) under the assumption that data were missing at random (MAR). The MAR assumption implies that the probability of missingness is related to observed variables but not to the unobserved outcomes themselves. MI was conducted using predictive mean matching, incorporating baseline demographic and clinical variables (eg, age, sex, education level, baseline PPD, CAL, and PCR) as predictors. A total of 20 imputed datasets were generated and pooled using Rubin’s rules to account for uncertainty in the imputations. Results from MI were compared with those from complete-case and worst-case sensitivity analyses to assess the robustness of the findings.

### Data Privacy, Security, and Retention Policies

To ensure the confidentiality and security of participant data, several safeguards were implemented throughout the study. The DentalMonitoring platform, used for remote intraoral monitoring, uses advanced encryption protocols to protect personal health data during both storage and transmission. Although specific technical details of the encryption standards are proprietary and not publicly disclosed, the platform complies with international information security standards.

All participants were fully informed of the data handling processes, including the collection, transmission, storage, and potential sharing of their data with the platform provider. Written informed consent was obtained before study enrollment, and participants were explicitly informed that their data would not be used for secondary purposes without additional consent. The consent process also ensured that participants understood their right to withdraw from the study and request deletion of their data at any time.

Personal data and intraoral photographs were retained only for the period required to complete data analysis and fulfill the research objectives. Specifically, all identifiable data were securely deleted within 3 months of study completion, in accordance with the institution’s data governance policies and ethical guidelines. The deletion process followed secure digital data disposal protocols to ensure irreversible removal, and no backup copies were retained following deletion.

The DentalMonitoring platform is certified under the French Health Data Hosting framework, which ensures compliance with national health data regulations, including physical security, secure backups, system redundancy, and real-time monitoring. In addition, the platform holds ISO/IEC 27001 certification for information security management and adheres to both the Health Insurance Portability and Accountability Act and the General Data Protection Regulation requirements. Security features include multifactor authentication, role-based access control, encrypted data processing, and regular independent security audits.

### Ethical Considerations

This study was approved by the Institutional Review Board of Kaohsiung Medical University Hospital, Kaohsiung, Taiwan (KMUHIRB-F(II)-20200059), and registered at ClinicalTrials.gov (NCT06083649). Written informed consent was obtained from all participants or their legal guardians before enrollment. Participant privacy and confidentiality were strictly protected, and all collected data were anonymized prior to analysis. To ensure participant welfare, the study covered both clinical examination fees and registration fees for periodontal treatment, without providing any additional financial compensation.

## Results

### Recruitment

**Figure 1.**
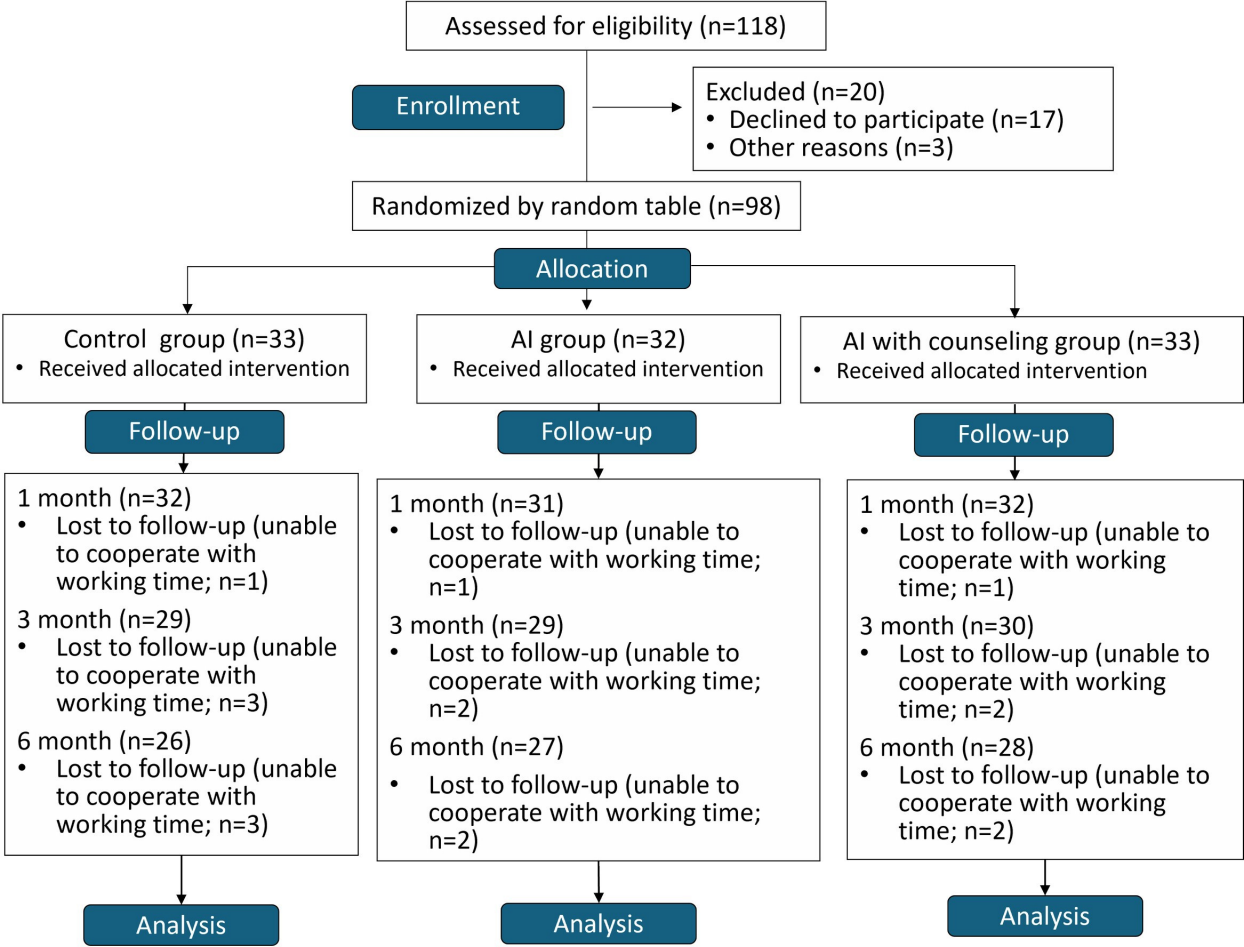
CONSORT-EHEALTH (Consolidated Standards of Reporting Trials of Electronic and Mobile Health Applications and Online Telehealth) flowchart of participant recruitment. An intention-to-treat analysis was used to analyze and compare groups. AI: artificial intelligence.

Figure 1 presents the CONSORT-EHEALTH (Consolidated Standards of Reporting Trials of Electronic and Mobile Health Applications and Online Telehealth [27]) flowchart of patient recruitment for the present randomized controlled trial. In total, 26 (78.8%), 27 (84.4%), and 28 (84.8%) patients in the control, AI, and AIHC groups, respectively, completed the study at all time points. An intention-to-treat analysis was used, meaning that all participants were analyzed in the groups to which they were originally assigned, with no crossover or reassignment during the study period.

Dropout analysis revealed no significant differences in baseline characteristics between participants who completed the study and those who dropped out at each follow-up (*P*>.05). To specifically evaluate whether educational attainment was associated with attrition, we categorized education into three levels: (1) below senior high school, (2) university, and (3) graduate school. A chi-square test indicated no statistically significant differences in educational distribution between completers and those lost to follow-up, suggesting that dropout was not related to education level.

The control group experienced a dropout rate exceeding 20%. To evaluate the impact of attrition on study outcomes, a sensitivity analysis assuming no improvement in periodontal parameters among dropouts was performed. The primary outcome remained statistically significant under this worst-case scenario, supporting the robustness of our findings. In addition, MI analyses were conducted to address missing data under the MAR assumption. Results from the imputed datasets were consistent with the primary analyses, further confirming the stability and reliability of the intervention effect.

### Baseline Characteristics

Table 1 presents the baseline information for the 3 groups. No significant intergroup differences were discovered at baseline regarding age, sex, or educational level (all *P*>.05).

**Table 1.** Baseline information of patients with periodontitis.

Variable	Control group (n=33)	AI^a^ group (n=32)	AIHC^b^ group (n=33)		*P* value
Age^c^ (years), mean (SD)	54.5 (9.1)	49.4 (7.7)	51.3 (8.7)		.05
Sex^c^, n (%)					.42
Male	14 (42.4)	17 (53.1)	12 (36.4)		
Female	19 (57.6)	15 (46.9)	21 (63.6)		
Education level^c^, n (%)					.47
Under senior high	11 (33.3)	9 (28.1)	11 (33.3)		
University	14 (42.4)	11 (34.4)	13 (39.4)		
Graduate school	8 (24.2)	12 (37.5)	9 (27.3)		

a AI: artificial intelligence.

b AIHC: artificial intelligence–assisted health counseling.

c Age was analyzed using one-way ANOVA, and sex and educational level were analyzed using the chi-square test.

### Interventions Effects on Threat Appraisal, Coping Appraisal, Self-Care Behavior, Dental Plaque, PPD, and CAL From Baseline to Follow-Up

**Figure 2.**
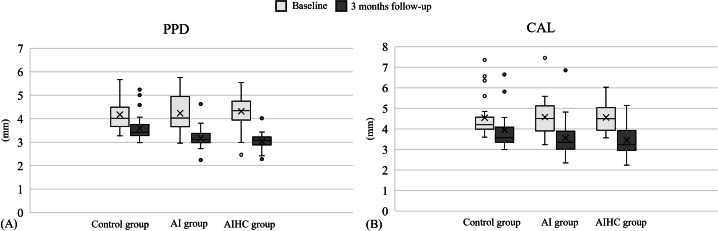
Boxplots of periodontal parameters by group and time: (A) probing pocket depth (PPD); (B) clinical attachment level (CAL). Each boxplot displays the distribution of values at baseline and 3-month follow-up across the 3 groups (control, artificial intelligence [AI], and artificial intelligence–assisted human counseling [AIHC]). The boxes represent the IQR, with the horizontal line inside each box indicating the median. The whiskers extend to the minimum and maximum values within 1.5 × IQR. Data points outside this range are plotted as individual outliers.

Table 2 summarizes the distributions and time trends of threat appraisal, coping appraisal, self-care behavior, PCR, PPD, and CAL from baseline to follow-up by treatment group. Significant time trends were observed for self-care behavior, PCR, PPD, and CAL across all groups (all *P* for trend <.001). Figures 2A and B show boxplots of PPD and CAL at baseline and 3 months, illustrating reductions in both parameters in the AI and AIHC groups, with narrower IQRs, lower medians, and fewer outliers at follow-up.

**Table 2.** Distributions and changes from baseline to follow-up in threat appraisal, coping appraisal, self-care behavior, plaque control record (PCR), probing pocket depth (PPD), and clinical attachment level (CAL) among patients with periodontitis by treatment group.

Variable	Control group	AI^a^ group	AIHC^b^ group
Threat appraisal (range 2‐20; mean [SD])			
Time 0^c^	17.8 (2.7)	17.0 (2.7)	17.7 (2.8)
Time 1^d^	18.5 (2.6)	18.2 (2.5)	18.3 (2.3)
Time 2^e^	19.1 (1.8)	18.6 (2.2)	19.3 (1.3)
Time 3^f^	18.9 (2.5)	19.4 (1.4)	18.9 (2.1)
*P* for time trend^g^	.45	.83	.78
Coping appraisal (range 2‐20; mean [SD])			
Time 0	17.3 (3.0)	17.4 (2.7)	17.5 (2.5)
Time 1	17.6 (2.9)	18.3 (2.1)	18.2 (2.5)
Time 2	18.3 (2.4)	18.2 (1.8)	18.6 (2.0)
Time 3	18.5 (2.8)	18.7 (1.5)	18.6 (2.0)
*P* for time trend^g^	.51	.07	.63
Self-care behavior (range 0‐5; mean [SD])			
Time 0	2.0 (1.1)	2.0 (1.3)	2.2 (1.1)
Time 1	3.3 (1.2)	3.5 (1.3)	4.0 (0.9)
Time 2	3.2 (1.3)	3.7 (1.0)	4.2 (0.7)
Time 3	3.2 (1.1)	4.1 (0.8)	4.4 (0.8)
*P* for time trend	<.001	<.001	<.001
PCR^h^ (%; mean [SD])			
Time 0	67.0 (14.9)	69.8 (18.2)	73.1 (14.2)
Time 1	47.9 (18.0)	52.0 (17.4)	50.2 (17.7)
Time 2	48.5 (17.3)	43.2 (14.8)	44.7 (14.7)
Time 3	49.2 (19.3)	38.7 (16.6)	32.2 (14.5)
*P* for time trend	<.001	<.001	<.001
PPD^i^ (mm; mean [SD])			
Time 0	4.3 (0.6)	4.3 (0.8)	4.3 (0.7)
Time 2	3.6 (0.6)	3.2 (0.5)	3.0 (0.4)
*P* for time trend	<.001	<.001	<.001
CAL^j^ (mm; mean [SD])			
Time 0	4.6 (1.0)	4.7 (0.9)	4.6 (0.6)
Time 2	4.0 (1.0)	3.6 (0.9)	3.4 (0.8)
*P* for time trend	<.001	<.001	<.001

a AI: artificial intelligence.

b AIHC: artificial intelligence–assisted health counseling.

c Time 0: baseline.

d Time 1: one-month follow-up.

e Time 2: three-month follow-up.

f Time 3: six-month follow-up.

g Due to nonnormality, the time trend was assessed using a logarithmically transformed variable.

h PCR: plaque control record.

i PPD: probing pocket depth.

j CAL: clinical attachment level.

Table 3 presents the proportions and time trends of self-care behaviors from baseline to follow-up by treatment group. In both the AI and AIHC groups, all 5 behaviors—brushing teeth more than 3 times a day, brushing for more than 3 minutes, using the Bass method, using an interdental brush, and using a soft-bristled toothbrush—showed significant positive trends over time (all *P* values for trend <.05).

**Table 3.** The proportions and time trends of self-care behaviors from baseline to follow-up among patients with periodontitis by treatment groups.

Variable	Control group	AI^a^ group	AIHC^b^ group
Brushing teeth more than 3 times a day, n (%)			
Time 0^c^	11 (33.3)	11 (34.4)	11 (33.3)
Time 1^d^	13 (40.6)	15 (48.4)	13 (40.6)
Time 2^e^	14 (48.3)	20 (69.0)	18 (60.0)
Time 3^f^	14 (53.8)	18 (66.7)	16 (57.1)
*P* for time trend	.04	<.001	<.001
Brushing teeth for over 3 minutes, n (%)			
Time 0	19 (57.6)	17 (53.1)	20 (60.6)
Time 1	27 (84.4)	22 (71.0)	30 (93.8)
Time 2	18 (62.1)	26 (90.0)	29 (96.6)
Time 3	18 (69.2)	22 (81.5)	27 (96.4)
*P* for time trend	.45	.002	<.001
Bass brushing method, n (%)			
Time 0	6 (18.2)	9 (28.1)	6 (18.2)
Time 1	22 (68.8)	27 (87.1)	26 (81.3)
Time 2	17 (58.6)	19 (65.5)	28 (93.3)
Time 3	13 (50.0)	25 (92.6)	26 (92.9)
*P* for time trend	.007	<.001	<.001
Interdental brushing, n (%)			
Time 0	9 (27.3)	8 (25.0)	7 (21.2)
Time 1	17 (53.1)	19 (61.3)	23 (71.9)
Time 2	18 (62.1)	18 (62.1)	22 (73.3)
Time 3	15 (57.7)	20 (74.1)	23 (82.1)
*P* for time trend	.004	<.001	.004
Using a soft-bristled toothbrush, n (%)			
Time 0	23 (69.7)	20 (62.5)	26 (78.8)
Time 1	28 (87.5)	24 (77.4)	32 (100.0)
Time 2	26 (86.7)	24 (82.8)	30 (100.0)
Time 3	26 (100.0)	27 (100.0)	28 (100.0)
*P* for time trend	.001	<.001	<.001

a AI: artificial intelligence.

b AIHC: artificial intelligence–assisted health counseling.

c Time 0: baseline.

d Time 1: one-month follow-up.

e Time 2: three-month follow-up.

f Time 3: six-month follow-up.

### Between-Group Comparison of PM Scores, Self-Care Behavior, Dental Plaque, PPD, and CAL

**Figure 3.**
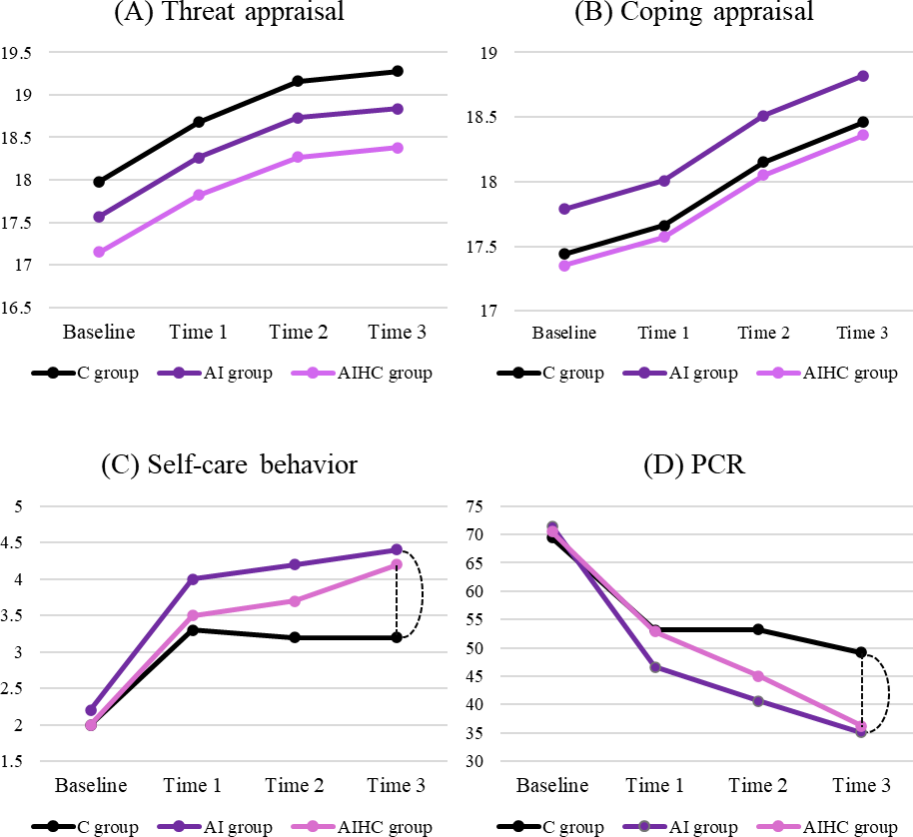
Mean distributions and interaction effect between group and time: (A) threat appraisal, (B) coping appraisal, (C) self-care behavior, (D) plaque control record (PCR). Values represent the adjusted means obtained from generalized estimating equation models adjusted for age, sex, and educational level. Dashed lines indicate significant differences between the artificial intelligence (AI) and artificial intelligence–assisted human counseling (AIHC) groups and the control group (*P*<.05). Time 1: 1-month follow-up, Time 2: 3-month follow-up, and Time 3: 6-month follow-up.

Table 4 summarizes the main and interaction effects of the intervention on PM scores, behaviors, and PCR, while Figure 3 illustrates the longitudinal changes in scores. A significant time effect was observed for threat appraisal at 1 month (adjusted mean ratio [aMR] 1.04, 95% CI 1.01‐1.07), 3 months (aMR 1.07, 95% CI 1.03‐1.11), and 6 months (aMR 1.07, 95% CI 1.03‐1.12); and for coping appraisal at both 3 months (aMR 1.04, 95% CI 1.00‐1.08) and 6 months (aMR 1.06, 95% CI 1.02‐1.10). For self-care behavior at 1 month (adjusted regression coefficient [aβ] 0.50, 95% CI 0.33‐0.66), 3 months (aβ 0.56, 95% CI 0.29‐0.64), and 6 months (aβ 0.44, 95% CI 0.26‐0.61); and for PCR at both 1 month (aβ −17.92, 95% CI −24.98 to −10.86), 3 months (aβ −20.44, 95% CI −27.71 to −13.18), and 6 months (aβ −18.17, 95% CI −25.29 to −11.04), significant time effects were also observed. Significant interaction effects were observed at 6 months: the AI group had greater improvements in self-care behavior (aβ 0.32, 95% CI 0.07‐0.57) and PCR (aβ −13.28, 95% CI −23.81 to −2.75); the AIHC group also showed improvements in self-care behavior (aβ 0.29, 95% CI 0.04‐0.53) and PCR (aβ −23.22, 95% CI −33.51 to −12.93) compared to the control group. The statistical analysis showed no significant group-by-time interaction for threat appraisal and coping appraisal at any time point (Figure 3A and 3B). In contrast, significant interaction effects were observed for self-care behavior and PCR at 6 months, with both the AI and AIHC groups showing greater improvements than the control group (all *P* values <.05; Figure 3C and 3D), indicating that AI-assisted monitoring led to superior long-term behavioral engagement and plaque control.

**Table 4.** Main and interaction effects of group and time on protection motivation scores, behaviors, and plaque control record (PCR) among patients with periodontitis.

Variables	Threat appraisal		Coping appraisal		Self-care behavior		PCR^a^	
	aMR^b^^c^ (95% CI)	*P* value	aMR^c^ (95% CI)	*P* value	aβ^d^^c^ (95% CI)	*P* value	aβ^c^ (95% CI)	*P* value
Group effect								
Control	Ref^e^	—^f^	Ref	—	Ref	—	Ref	—
AI^g^	0.98 (0.93 to 1.03)	.39	1.02 (0.97 to 1.07)	.46	0.00 (−0.19 to 0.19)	.98	2.86 (−5.40 to 11.11)	.50
AIHC^h^	0.95 (0.91 to 1.00)	.08	0.99 (0.94 to 1.05)	.84	0.07 (−0.11 to 0.26)	.45	6.01 (1.62 to 13.80)	.12
Time effect								
Time 0^i^	Ref	—	Ref	—	Ref	—	Ref	—
Time 1^j^	1.04 (1.01 to 1.07)	.01	1.01 (0.98 to 1.04)	.39	0.50 (0.33 to 0.66)	<.001	−17.92 (−24.98 to −10.86)	<.001
Time 2^k^	1.07 (1.03 to 1.11)	.001	1.04 (1.00 to 1.08)	.03	0.56 (0.29 to 0.64)	<.001	−20.44 (27.71 to −13.18)	<.001
Time 3^l^	1.07 (1.03 to 1.12)	.001	1.06 (1.02 to 1.10)	.01	0.44 (0.26 to 0.61)	<.001	−18.17 (−25.29 to −11.04)	<.001
Interaction effect (group × time points)								
Control × Time 0	—	—	—	—	Ref	—	Ref	—
AI × Time 1	—	—	—	—	−0.00 (−0.24 to 0.23)	.98	1.28 (−8.83 to 11.39)	.80
AI × Time 2	—	—	—	—	0.14 (−0.10 to 0.39)	.25	−3.49 (−13.86 to 6.87)	.51
AI × Time 3	—	—	—	—	0.32 (0.07 to 0.57)	.01	−13.28 (−23.81 to −2.75)	.01
AIHC × Time 1	—	—	—	—	0.12 (−0.11 to 0.36)	.31	−5.00 (14.98 to 4.97)	.33
AIHC × Time 2	—	—	—	—	0.23 (−0.01 to 0.48)	.06	−5.64 (−15.82 to 4.53)	.28
AIHC × Time 3	—	—	—	—	0.29 (0.04 to 0.53)	.02	−23.22 (−33.51 to −12.93)	<.001

a PCR: plaque control record.

b aMR: adjusted mean ratio.

c Since the outcome is nonnormally distributed, the GEE model was specified with a gamma distribution and a log link function. The MR represents the ratio of the investigated group’s mean outcome to that of the control group. In contrast, β represents the difference in mean outcomes between the investigated group and the control group. All GEE models were adjusted for age, sex, and educational level.

d aβ: adjusted regression coefficient.

e Ref: reference group.

f Not applicable.

g AI: artificial intelligence.

h AIHC: artificial intelligence–assisted health counseling.

i Time 0: baseline.

j Time 1: one-month follow-up.

k Time 2: three-month follow-up.

l Time 3: six-month follow-up.

**Figure 4.**
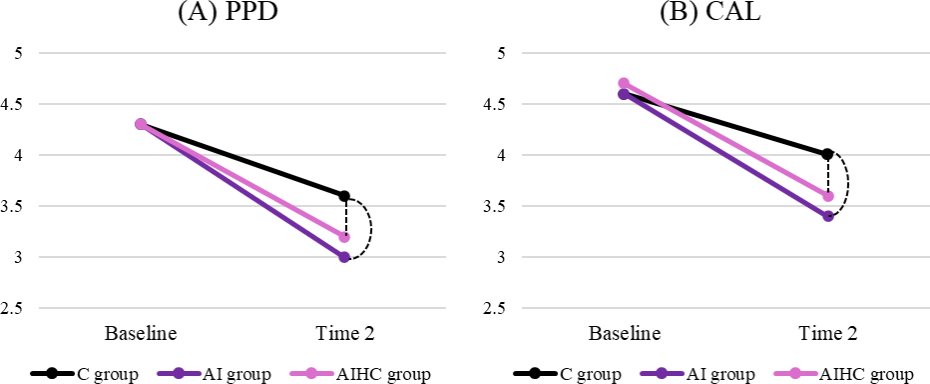
Mean distributions and interaction effect between group and time: (A) probing pocket depth (PPD) and (B) clinical attachment level (CAL). Values represent the adjusted means obtained from generalized estimating equation models adjusted for age, sex, and educational level. Dashed lines indicate significant differences between the artificial intelligence (AI) and artificial intelligence–assisted human counseling (AIHC) groups and the control group (*P*<.05). Time 2: 3-month follow-up.

Table 5 summarizes the main and interaction effects of the intervention on periodontal parameters, while Figure 4 illustrates the longitudinal changes in score. At 3 months, significant time effects were observed for both PPD (aβ −0.70, 95% CI −0.93 to −0.47) and CAL (aβ −0.66, 95% CI −0.93 to −0.40). Significant interaction effects at 3 months indicated that both the AI and AIHC groups showed greater reductions in PPD (AI: aβ −0.43, 95% CI −0.76 to −0.10; AIHC: aβ −0.55, 95% CI −0.88 to −0.23) and CAL (AI: aβ −0.44, 95% CI −0.82 to −0.07; AIHC: aβ −0.51, 95% CI −0.88 to −0.14) compared with the control group. Figure 4A and 4B further illustrate these interaction effects, showing steeper declines in PPD and CAL for both AI-based intervention groups compared with the control group (all *P*<.05), confirming that the AI-assisted approaches achieved greater clinical improvements during the first 3 months.

**Table 5.** Main and interaction effects of group and time on periodontal parameters among patients with periodontitis.^a^

Variables	PPD^b^			CAL^c^		
	aβ^d^ (95% CI)		*P* value	aβ (95% CI)		*P* value
Group effect						
Control	Ref^e^		—	Ref		—
AI^f^	0.02 (−0.30 to 0.35)		.89	0.06 (−0.44 to 0.55)		.82
AIHC^g^	−0.00 (−0.31 to 0.35)		.99	−0.00 (−0.44 to 0.43)		.99
Time effect						
Time 0^h^	Ref		—	Ref		—
Time 2^i^	−0.70 (−0.93 to −0.47)		<.001	−0.66 (−0.93 to −0.40)		<.001
Interaction effect (group × time points)						
Control × Time 0	Ref		—	Ref		—
AI × Time 2	−0.43 (−0.72 to −0.14)		.004	−0.44 (−0.82 to −0.07)		.02
AIHC × Time 2	−0.55 (−0.88 to −0.23)		.001	−0.51 (−0.88 to −0.14)		.007

a All generalized estimating equation (GEE) models were adjusted for age, sex, and educational level.

b PPD: probing pocket depth.

c CAL: clinical attachment level.

d aβ: adjusted regression coefficient.

e Ref: reference group.

f AI: artificial intelligence.

g AIHC: artificial intelligence–assisted health counseling.

h Time 0: baseline.

i Time 2: three-month follow-up.

## Discussion

### Principal Findings

This randomized controlled trial demonstrated that AI-assisted dental monitoring—with or without additional health counseling—significantly improved clinical and behavioral outcomes in patients with periodontitis. At the 3-month follow-up, both intervention groups showed greater reductions in PPD and CAL compared with the control group. By 6 months, both intervention groups exhibited significantly better self-care behaviors and lower PCR scores than the control group. Regarding PM constructs, although significant improvements over time were observed for both threat appraisal and coping appraisal across all groups, no significant group-by-time interaction was detected. This indicates that while AI-assisted interventions did not differentially impact these psychological constructs compared with standard care, participants overall experienced increased awareness of risk and enhanced confidence in managing their oral health. The improvements in motivation may be partly attributed to standardized oral health education provided to all groups. Together, these findings suggest that AI-assisted monitoring effectively enhances clinical and behavioral outcomes beyond standard education, potentially through mechanisms other than changes in PM constructs.

According to Lindhe et al [28], nonsurgical periodontal therapy typically yields a 1‐2 mm reduction in PPD and a 0‐1 mm gain in CAL at sites with moderate baseline depths. In our study, the observed improvements (~1.1‐1.3 mm in PPD and ~1.0‐1.2 mm in CAL) fall within or slightly exceed these clinical expectations, suggesting clinically meaningful changes. Moreover, significant group-by-time interaction effects indicated that participants receiving AI-assisted monitoring (AI and AIHC groups) achieved greater reductions in PPD and CAL than those receiving standard care alone. These greater reductions in PPD and CAL are consistent with our previous studies [14,15], where AI-assisted intervention groups also showed superior periodontal outcomes during follow-up. These clinical improvements were accompanied by sustained enhancements in self-care behaviors and plaque control, supporting the notion that behavioral change contributed to improved periodontal outcomes. Overall, these findings highlight the potential added value of AI-assisted monitoring as an adjunct to nonsurgical periodontal therapy, supporting its role in enhancing clinical outcomes beyond conventional approaches.

The patients in the AI and AIHC groups demonstrated significantly greater improvements in self-care behaviors and PCR at the 6-month follow-up. This suggests that the AI-based smartphone platform for at-home oral hygiene monitoring is more effective than traditional oral health education alone, likely due to AI’s ability to provide timely reminders and ongoing engagement. These results are consistent with those of a study on a PM theory–based intervention in which significant improvements were discovered in oral health behaviors, self-efficacy, and perceived disease severity following the intervention [29]. In this study, the participants could view scanned images of their mouths, taken at home on their smartphones, to assess their oral hygiene and inflammation status at any time. The AI reminders also motivated the participants, improving their oral self-care behaviors and reducing their amount of dental plaque. At the 6-month follow-up, the AI and AIHC groups in this study also exhibited significant improvements in terms of brushing teeth 3 or more times a day, brushing teeth for more than 3 minutes, using the Bass brushing method, interdental brushing, and using a soft-bristled toothbrush. These findings are consistent with those of a study that used a conversational AI system to promote health behavior changes [30].

This study found that at 6 months, threat appraisal scores increased by approximately 7%, and coping appraisal scores by about 6%, indicating sustained higher risk awareness and greater confidence in oral health management among participants. The absence of a significant group-by-time interaction may be explained by the standardized oral health instructions provided to all participants, including those in the control group, which likely enhanced risk perception and coping confidence across groups, thereby reducing between-group differences while supporting overall improvement. These findings are consistent with Bandura’s theory of self-efficacy and personal agency [31], which emphasizes that an individual’s belief in their ability to control their actions and environment is critical to motivation and behavioral change. The sustained improvement in both threat appraisal and coping appraisal suggests that the interventions strengthened participants’ self-efficacy, which in turn facilitated proactive self-care behaviors. Overall, these findings suggest that strengthening motivation can enhance self-efficacy, thereby facilitating better oral health outcomes.

### Limitations

This study has several limitations. First, due to the nature of the intervention, participant blinding was not feasible, which may introduce performance bias. Although clinical outcome assessors were blinded to group allocation to minimize detection bias, the statistician was not blinded during data analysis, representing a potential source of bias. Second, the Hawthorne effect may have influenced participants’ behavior, especially in the AI and AIHC groups who received more frequent monitoring and interaction. While this effect cannot be entirely ruled out, it reflects a common challenge in behavioral intervention studies and may indicate the real-world potential of digital health tools to enhance motivation and adherence. To mitigate bias, we included PCR as an objective outcome measure. Third, social desirability bias may have influenced the results; however, randomization helped minimize this risk. Fourth, the control group experienced >20% attrition, raising concerns of bias. We addressed this through a worst-case sensitivity analysis and MI under the MAR assumption. Both analyses supported the robustness of our findings, although missing data remain a limitation if the missingness mechanism differs from MAR. Future studies should aim to minimize dropout and explore alternative analytic methods. Fifth, while multiple comparisons were conducted between the AI, AIHC, and control groups, these were prespecified and hypothesis-driven rather than exploratory. Therefore, no additional adjustments for multiple testing were applied. Nonetheless, we acknowledge this as a methodological limitation and recommend cautious interpretation of the findings due to the potential increased risk of Type I error. Finally, the use of AI-based digital technology may limit the generalizability of our findings to populations with lower education, digital literacy, or socioeconomic status. Although our dropout analysis did not reveal a significant association between education level and attrition, accessibility challenges remain. To support user engagement regardless of literacy level, the intervention incorporated simplified visual interfaces, voice-guided prompts, and instructional videos to enhance usability and reduce participation barriers. However, broader implementation may require further adaptations such as multilingual content, culturally appropriate onboarding, community-based digital health facilitators, and tailored educational materials to ensure equitable access and applicability across underserved populations.

This study was conducted at a single center using the DentalMonitoring platform, which requires specific hardware such as a scan box and consumer-grade smartphones. While patients used their own smartphones, reducing equipment costs, the financial burden of acquiring additional devices and software licenses may limit accessibility, especially in lower-resource settings. Moreover, the reliance on digital technology necessitates a minimum level of digital literacy, posing challenges for populations with limited experience or access to such tools. Implementation in low-resource or underserved environments may require simplified user interfaces, culturally sensitive onboarding, and community support to improve feasibility and uptake. Future studies should explore cost-effectiveness and scalable models to broaden accessibility and ensure equitable benefits from AI-assisted periodontal care.

### Conclusions

This study demonstrated that AI-assisted monitoring significantly improved patients’ self-care behaviors, plaque control, and periodontal clinical outcomes compared to standard care. Both AI and AIHC groups showed greater increases in self-care behaviors and reductions in plaque levels at 6 months, as well as superior clinical improvements in PPD and CAL at 3 months. While PM constructs such as threat appraisal and coping appraisal increased similarly across all groups, the more pronounced behavioral and clinical benefits observed in the AI-assisted groups underscore the value of AI technologies in periodontal management. Incorporating AI-assisted monitoring into routine clinical practice may empower patients to engage more actively in their oral health care, facilitating more effective and patient-centered periodontal therapy. To promote broader adoption and health equity, future applications should prioritize user-friendly designs, multilingual support, and accessibility for populations with lower health or digital literacy.

## Supplementary material

10.2196/78211 Checklist 1 CONSORT-EHEALTH checklist (V 1.6.1).

## Abbreviations

AI: artificial intelligence
AIHC: artificial intelligence–assisted health counseling
aMR: adjusted mean ratio
aβ: adjusted regression coefficient
CAL: clinical attachment level
CONSORT-EHEALTH: Consolidated Standards of Reporting Trials of Electronic and Mobile Health Applications and Online Telehealth
GEE: generalized estimating equation
MAR: missing at random
MI: multiple imputation
PCR: plaque control record
PM: protection motivation
PPD: probing pocket depth
